# Linked administrative data can inform policy and practice: An academic - government research partnership in South Australia.

**DOI:** 10.23889/ijpds.v7i3.1965

**Published:** 2022-08-25

**Authors:** Rhiannon Pilkington, Alicia Montgomerie, Angela Gialamas, John Lynch

**Affiliations:** 1 School of Public Health, University of Adelaide, Australia; 2 School of Psychology, University of Adelaide, Australia; 3 School of Population Health Sciences, University of Bristol

## Objectives

To describe how the Better Evidence Better Outcomes Linked Data (BEBOLD) platform has been used to partner with government agencies to generate evidence to support service reform that contributes to improving outcomes for children and families in contact with the child protection system.

## Approach

Data was drawn from the BEBOLD platform, a whole-of-population linked de-identified administrative data platform for all children in South Australia born from 1991-2016 (n~500,000), as well as their parents. Data linked included birth registrations, perinatal statistics, child protection services, family health services, education, youth justice, housing and homelessness, emergency department presentations and hospitalisation, as well as adult drug and alcohol services.

## Results

We present case studies illustrating how the BEBOLD platform has been used to 1) investigate the descriptive epidemiology of child protection contact patterns with a focus on priority populations (e.g. young parents); 2) describe service interactions of child protection populations in a way that highlights prevention potential (e.g. the overlap between public housing and child protection); and 3) identify policy defined target populations to inform contracting of support services (e.g. estimate counterfactual reunification rates for a defined population to inform development of a Social Impact Bond).

## Conclusion

Using linked data to take an intelligent system view of potential priority populations can inform policy and service delivery in a resource restricted setting. Descriptive epidemiology of child protection contact patterns, population sizes, transitions, and characteristics can aid services to explore elements of service or system design including eligibility criteria.

